# Management of Cardiovascular Disorders in Patients with Noonan Syndrome: A Case Report

**Published:** 2017-10

**Authors:** Mohammad Rafie Khorgami, Maryam Moradian, Negar Omidi, Mohammad Yousef Aarabi Moghadam

**Affiliations:** 1 *Rajaie Cardiovascular Medical and Research Center, Iran University of Medical Sciences, Tehran, Iran.*; 2 *Primary Prevention Cardiovascular Research Center, Tehran Heart Center, Tehran University of Medical Sciences, Tehran, Iran.*

**Keywords:** *Noonan syndrome*, *Cardiovascular diseases*, *Pediatrics*, *Heart defects, congenital*

## Abstract

The Noonan syndrome is a rare disorder, one of whose major complications is cardiovascular involvement. A wide spectrum of congenital heart diseases has been observed in this syndrome. The most common cardiac disorder is pulmonary valve stenosis, which has a progressive nature. Hypertrophic cardiomyopathy is less common, but its morbidity and mortality rates are high. We herein introduce a 12-year-old boy with the typical findings of the Noonan syndrome. His symptoms began from infancy, and there was a gradual exacerbation in his respiratory and cardiac manifestations with age. The cardiac involvement included right ventricular outflow tract and pulmonary valve stenosis, hypertrophic cardiomyopathy, and subaortic valve stenosis. Due to the progressive course of the disease, surgical repair was done. Although the patient had a difficult postoperative period, his general condition improved and he was discharged. At 3 months’ follow-up, his symptoms showed improvement. Additionally, there was a reduction in the echocardiographic parameters of the outflow tract stenosis gradient as well as a significant improvement in the cardiac hemodynamic indices.

## Introduction

The Noonan syndrome is a rare disorder with diverse body organs involvement and one of the main organs that involved in this syndrome is cardiovascular system.^1^

The most common cardiac disorder is pulmonary valve stenosis, which has a progressive nature. Hypertrophic cardiomyopathy is less common. The Noonan syndrome is a genetic disorder that usually was observed as sporadic pattern but autosomal transmission also was seen. Because the cardiac diseases have progressive nature in Noonan syndrome development of cardiac symptoms and signs necessitate careful cardiac evaluation and prompt treatment.^1^^, ^^2^

## Case Report

A 12-year-old boy was admitted to our hospital due to shortness of breath, exertional dyspnea, and exercise intolerance. Based on his past medical history as an infant, the 1st signs had begun when he was 4 months old by bluish discoloration of the lips, lethargy, and poor feeding. His mother declared that her child suffered from inguinal hernia but that he had no other disease. He had another sibling, who was healthy. There was no family history of congenital cardiac disease or sudden cardiac death.

**Figure 1:**
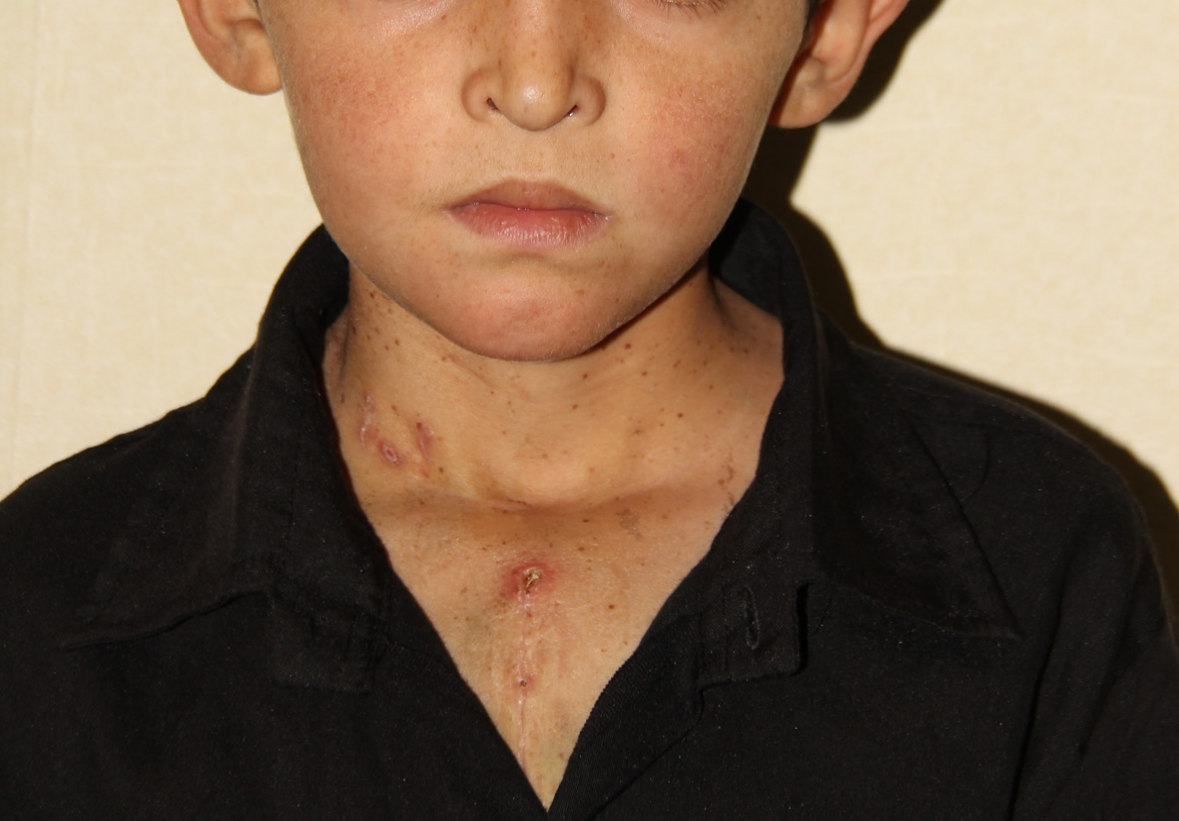
Dysmorphic facial features, characteristic of the Noonan syndrome: hypertelorism, downward slant of the palpebral fissures, inverted triangle-shaped head, and small chin.

Gradually as the patient grew up, his symptoms increased and mere usual childhood activities let to dyspnea and fatigue. On physical examination, short stature, failure to thrive, and chest wall deformity were noted. Additionally, he had special facial characteristics such as low-set ears, ptosis, hypertelorism, and low posterior hairline (Figure 1). Multiple lentigines were observed on his face, trunk, and extremities as well. Cardiac auscultation findings included normal 1st heart sound, single 2nd sound, and a grade 4/6 systolic murmur at the left sternal border.

**Figure 2:**
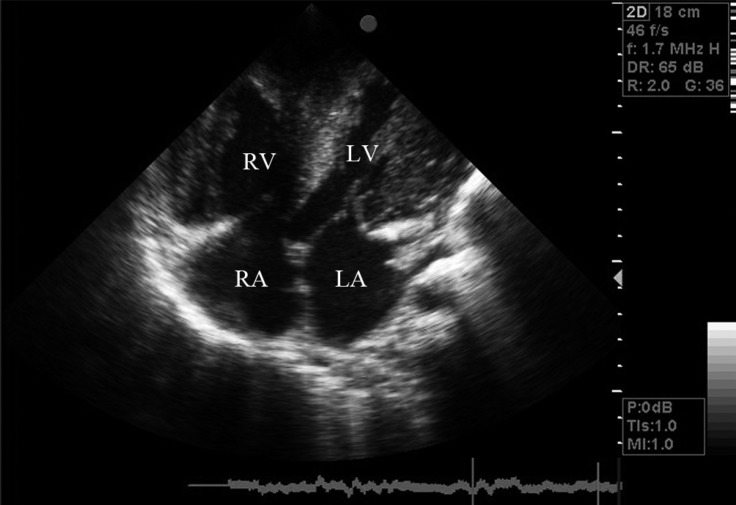
4-chamber view in echocardiography showed severe LV hypertrophy indicated hypertrophic cardiomyopathy.

**Figure 3:**
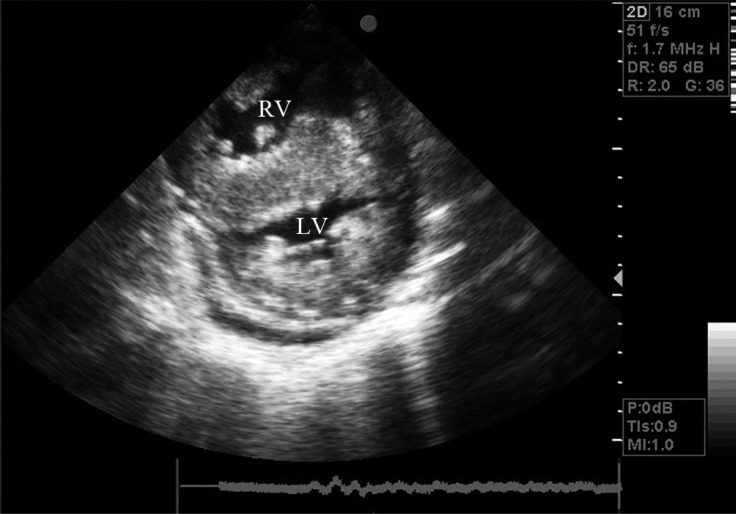
Echocardiography in short-axis view, showing concentric hypertrophic cardiomyopathy.

**Figure 4:**
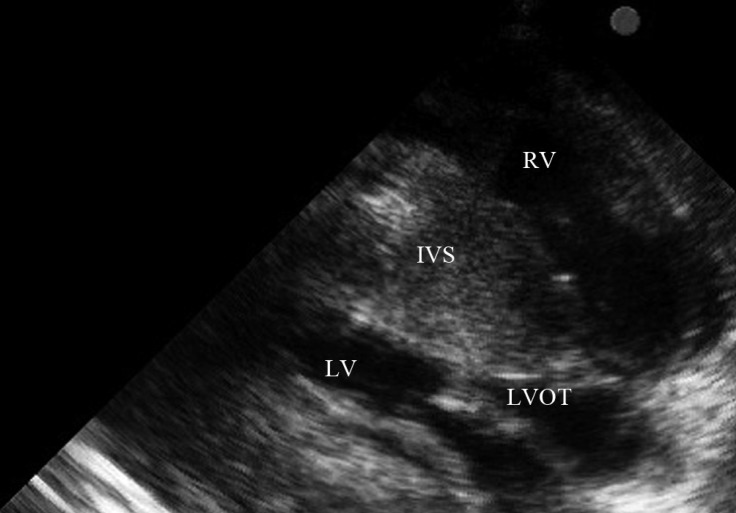
Long-axis view in echocardiography, showing severe concentric hypertrophic cardiomyopathy with significant subaortic obstruction.

RV, Right ventricle; LV, Left ventricle; LVOT, Left ventricular outflow tract; IVS, Interventricular septum

**Figure 5:**
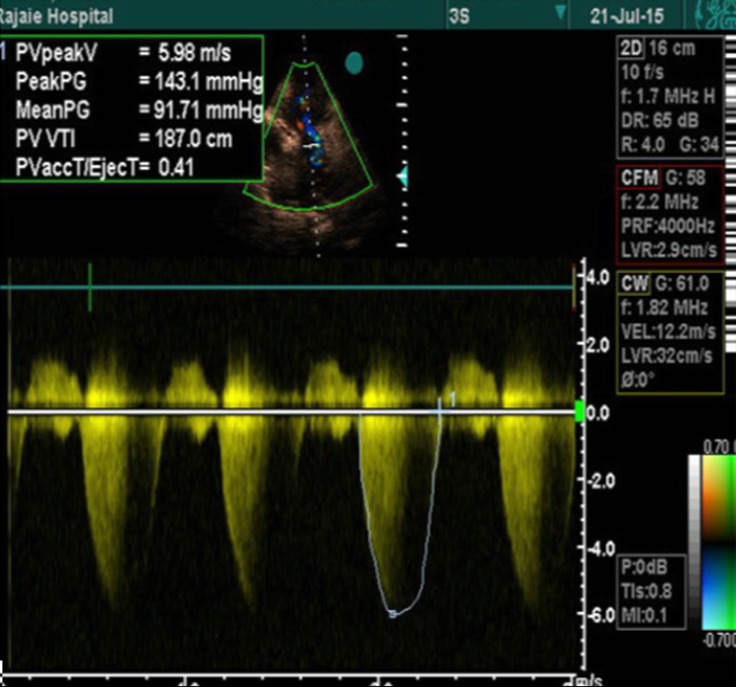
Pressure gradient via the pulmonary valve, measured by continuous Doppler echocardiography.

Chest X-ray revealed an increased cardiothoracic ratio and a decreased pulmonary vascular marking. The laboratory test was normal except for iron deficiency anemia (hemoglobin = 10.2 g/L). Electrocardiography (ECG) yielded evidence of biventricular hypertrophy, biatrial enlargement, and ST–T change. The family showed us another ECG trace of his infantile period, which also propounded hypertrophic cardiomyopathy (HCM). Medical treatment with propranolol and captopril was recommended. The patient, however, failed to consume these drugs regularly. Echocardiographic findings included severe left and right ventricular hypertrophy, severe mitral valve regurgitation, moderate dynamic subvalvular aortic stenosis with a 56 mmHg pressure gradient, severe tricuspid regurgitation, severe subvalvular and valvular pulmonary stenosis with a 150 mmHg pressure gradient via the pulmonary valve, and reduced left ventricular (LV) systolic (LV ejection fraction = 35%) and diastolic (impaired relaxation) function (Figures 2–5).

RV, Right ventricle, LV, Left ventricle; RA, Right atrium; LA, Left atrium

RV, Right ventricle, LV, Left ventricle

Due to severe pulmonary valve stenosis, percutaneous pulmonary balloon valvuloplasty was done 1 year before the present article was written, but there was no significant decrease in the pressure gradient via the pulmonary valve. Because the patient’s symptoms and signs were progressive and there was no proper response to drug therapy, he was considered for surgical repair. During surgery, via a right ventricular outflow tract incision, the outflow tract muscle was shaved and the pulmonary valve was repaired. Also, septomyomectomy was done in the left ventricular outflow tract (LVOT) region. After surgery, in the intensive care unit, the patient had a devastating postoperative course. A combination of low cardiac output, disseminated intravascular coagulation, and ischemic pattern due to HCM and frequent arrhythmia deteriorated his condition. His condition was further exacerbated by gastrointestinal bleeding and bleeding from the chest tube. Acidosis and electrolyte imbalance were corrected, and inotropes and broad-spectrum antibiotics were prescribed. He then developed junctional ectopic tachycardia, which was treated with intravenous amiodarone. Gradually, however, his general condition improved. At the time of his discharge, echocardiography revealed better LV systolic function (LV ejection fraction = 45%), reduced pressure gradient via the pulmonary valve (60 mmHg), and a 10 mmHg pressure gradient between the LVOT and the aortic valve. At 3 months’ follow-up, his functional class showed a significant improvement, as confirmed by the exercise test.

Our patient has a single major pediatric risk factor (maximum LV wall thickness ≥ 30 mm), which is class IIb for the implantation of the implantable cardioverter-defibrillator (ICD). Nonetheless, in the future, he may be candidated for ICD implantation and may, as such, need very careful observation.

## Discussion

The Noonan syndrome is a rare but well-recognized genetic syndrome, and cardiovascular disorder is one of its major complications. The incidence of the Noonan syndrome is between 1,000 and 2,500 in live births.^3^^-^^5^ The Noonan syndrome is a heterogeneous genetic syndrome, but mutation in the PTPN11 gene on chromosome 12 has been found in approximately 50% of the patients.^6^ The syndrome usually has a sporadic pattern, but autosomal dominant transition has also been reported.^7^ Owing to multiple organ involvement, a wide spectrum of symptoms and signs may occur in this syndrome (Table 1)^8^^, ^^9^ and most patients are referred for medical management during infancy and childhood.

Most patients with the Noonan syndrome have cardiac abnormalities, which predominantly present as congenital heart defects, cardiomyopathy, arrhythmia, or a combination of all. Pulmonary valve stenosis is the most common congenital heart defect.^10^ The pulmonary valve may be dysplastic and malformed, and it may be accompanied by subvalvular stenosis.^11^ In a study by Marino et al.^12^ on 136 patients with the Noonan syndrome, pulmonary stenosis was reported in 39% of the patients. Although HCM in the Noonan syndrome is less common, its morbidity rate is high. The association between HCM in the Noonan syndrome and sudden cardiac death and heart failure necessitates an exact evaluation. HCM presents in earlier age often during infancy, and LVOT obstruction is also common (average gradient = 32 mmHg).^13^^, ^^14^

**Table 1:** Clinical features of the Noonan syndrome

Facial dysmorphism: hypertelorism, downward slant of palpebral fis- sures, epicanthal folds, triangular facies, small chin, low-set and/or pos- teriorly rotated ears, webbed neck with low posterior hairline Cardiovascular: pulmonic stenosis and dysplasia, hypertrophic cardio- myopathy, secundum atrial septal defects, atrioventricular septal defects, tetralogy of Fallot Skeletal: pectus excavatum and/or carinatum, cubitus valgus, scoliosis Hematology: bleeding diathesis, leukemia Development: delay, attention deficit/hyperactivity disorder Oral/Dental: articulation difficulty, high arched palate, malocclusion Cryptorchidism Skin: dystrophic nails, follicular keratosis, hyperelastic skin, multiple lentigines

In our patient, the presenting sign was cardiac involvement, beginning during infancy. A combination of pulmonary valve stenosis, a usual cardiac sign in the Noonan syndrome, and HCM rendered the management of this patient very difficult. We tried in vain to eliminate the pulmonary valve stenosis with balloon valvuloplasty, which seemed reasonable given the dysplastic valve morphology and stenosis in the subvalvular region. On the other hand, HCM with its progressive nature led to severe diastolic and systolic dysfunction, which together with right ventricular systolic and diastolic dysfunction lessened the functional class and exercise tolerance and-in short-caused an almost intolerable condition for the child. In a study by Poterucha et al.,^15^ surgical myectomy in patients with the Noonan syndrome and HCM with LVOT obstruction decreased the LVOT gradient and the New York Heart Association’s class. These results are similar to those reported in patients with nonsyndromic HCM. 

Although we expected the patient’s bad course in the early postoperative period, the addition of sepsis to low cardiac output state after surgery made this condition even worse than was expected. With appropriate treatment, the patient’s cardiac function and general condition gradually improved. The next step in the management of this patient is the decision-making for ICD insertion.^16^

## Conclusion

The cardiovascular involvement in the Noonan syndrome has a progressive and often life-threatening course. We recommend a comprehensive evaluation for cardiovascular disorders, especially pulmonary valve involvement and HCM, in children with the Noonan syndrome. Repair of cardiovascular disorders in earlier age before severe injury to the right ventricular and LV myocardium may prevent post-surgical complications and long-term hospitalization.

## Notes:

***This paper should be cited as: ***Khorgami MR, Moradian M, Omidi N, Yousef M. Management of Cardiovascular Disorders in Patients with Noonan Syndrome. J Teh Univ Heart Ctr 2017;12(4):184-187.
